# Fascioliasis associated with chronic cholecystitis in a woman from Sistan and Baluchestan province, a non-endemic region in Southeastern Iran

**DOI:** 10.1186/s12879-023-08310-z

**Published:** 2023-05-19

**Authors:** Mohammad Shafiee, Saeid Nasibi, Mohammad Reza Lashkarizadeh, Majid Fasihi Harandi

**Affiliations:** 1 Research Center for Hydatid Disease in Iran, Dept of Surgery, Afzalipour Medical Center, School of Medicine, Kerman University of Medical Sciences, Kerman, Iran; 2 Research Center for Hydatid Disease in Iran, Dept of Parasitology, Afzalipour School of Medicine, Kerman University of Medical Sciences, Kerman, Iran

**Keywords:** Fasciolosis, *Fasciola hepatica*, Chronic cholecystitis, Endosonography, Case report

## Abstract

**Background:**

Fascioliasis, caused by *Fasciola hepatica*, is a neglected zoonotic food-borne trematodiasis. The Caspian littoral in northern Iran is endemic for the disease, and human fascioliasis is well-known in that region. In the present study, we report the diagnosis, identification, and clinical management of a human case of fascioliasis associated with common bile duct (CBD) obstruction from a non-endemic remote area in southeastern Iran.

**Case presentation:**

A 42-year-old female was admitted to Afzalipour Medical Center hepatobiliary surgery ward in Kerman with abdominal pain for the past three months. Dilated biliary tract and an ill-defined mass in CBD were reported in abdominal ultrasonography and magnetic resonance cholangiopancreatography, respectively. During distal CBD operation, nine leaf-like motile flatworms were isolated. A morphological study confirmed all the isolates as *Fasciola*, and further molecular investigations, identified the flukes as *F. hepatica* using both pepck multiplex PCR and cox1 sequencing.

**Conclusion:**

Molecular and morphological findings of the study indicated the presence of human fascioliasis in the southeastern province of Sistan and Baluchestan in Iran. Fascioliasis is among the etiologies of chronic cholecystitis, and physicians should consider chronic cholecystitis associated with fascioliasis in the differential diagnosis. In the present report, endoscopic ultrasound was usefully applied for the accurate diagnosis of biliary fasciolosis.

**Supplementary Information:**

The online version contains supplementary material available at 10.1186/s12879-023-08310-z.

## Background

Fascioliasis is a zoonotic food-borne parasitic disease, mainly caused by the liver fluke, *Fasciola hepatica*, and *F. gigantica.* Human and livestock infection occurs by ingestion of metacercariae during the consumption of contaminated raw vegetables or drinking water. In humans, maturation of metacercaria into the adult fluke takes approximately 3 to 4 months [1]. Metacercariae excyst and penetrate through the gut wall into the abdominal cavity and migrate toward the liver and bile ducts, causing biliary fibrosis or bile duct obstruction and dilatation [2].

Fascioliasis can cause a spectrum of clinical presentation in the acute and chronic phases of the infection including gastrointestinal symptoms, abdominal pain in the right upper quadrant, hepatomegaly, mild hepatitis, chronic cholecystitis, cholangitis, fatigue, anemia, pancreatitis, and weight loss. Biliary tract obstruction by *F. hepatica* is rare and surgical intervention is recommended [3].

Human fascioliasis is well-known in Iran after two major epidemics in 1989 and 1999 in the northern province of Gilan. Subsequently, World Health Organization (WHO) considered Iran among the six most infected countries with serious concerns about *Fasciola* infection [4]. In Iran, the disease is most frequently seen in the humid regions of northern Gilan province, and few cases have been rarely reported from other parts of the country e.g., Yasuj, Lorestan, and Kermanshah in the west [5–7]. Moderate temperature, rainfall during the year, a high population of Lymnaeid freshwater snails as intermediate hosts, and large pastures for ruminants provide appropriate conditions for the transmission of fascioliasis in this area. This study presents a human case of fascioliasis associated with CBD obstruction from Saravan County, Sistan and Baluchestan province in the southeast of Iran.

## Case presentation

**Fig. 1:**
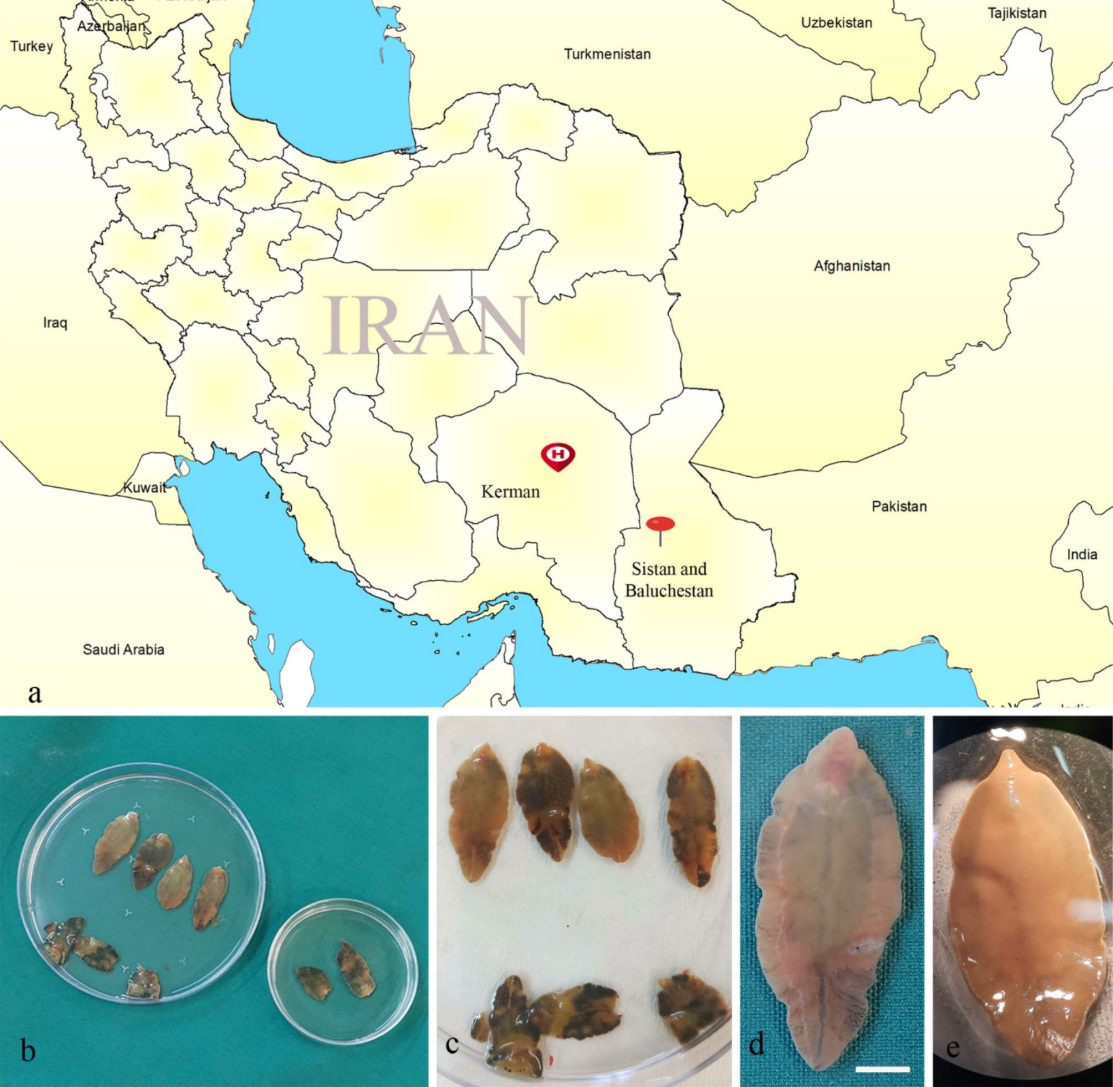
(**a**) Geographical description of the patient’s location in the Sistan and Baluchestan province, southeastern Iran (red pin) and Afzalipour Medical Center in Kerman (red H symbol). (**b-e**) Adult worms of *Fasciola hepatica* extracted from distal parts of the common bile duct of a 42-year-old female patient. scale bar: 5 mm

A 42-year-old female with abdominal pain in the right upper quadrant (RUQ) for the past three months, was admitted to Afzalipour Medical Center hepatobiliary surgery ward in Kerman. The patient was a housewife living in rural areas of the southeastern province of Sistan and Baluchestan in Iran. (Fig. 1a). She reported very little raw vegetable consumption since she has been suffering from indigestion. No history of fever, weight loss, urticaria, diarrhea, jaundice, and cough was described. Remarkable laboratory findings include mild leukocytosis (WBC: 5700/mm3), anemia (RBC: 3.64 × 10^6^/mm3, Hb: 8.7 g/dl, HCT: 28.5%, MCV: 78.3 fl.), and hypoglycemia (FBS = 77 mg/dl). Other serum factors like bilirubin, gamma-glutamyl transpeptidase (GGT), aspartate aminotransferase (AST), alanine aminotransferase (ALT), and alkaline phosphatase (ALP) were within normal range. A history of hypothyroidism was recorded in the patient hospital documents.

In abdominal ultrasonography, a dilated biliary tract was reported. Magnetic Resonance Cholangiopancreatography (MRCP) revealed dilated Common Bile Duct (CBD) and an ill-defined mass in CBD. Subsequently, Endoscopic Ultrasound (EUS) was performed by linear echo-endoscope. EUS reported a hyperechoic parenchyma in the liver seen without intrahepatic stone and normal left adrenal. CBD was dilated and measured at 15 mm and 8 mm in proximal and distal parts, respectively. CBD contained a large 14 mm in diameter mass lesion. Gallbladder had a lot of sludge and micro lithiasis. In the pancreas, dilated pancreatic duct was measured at 4 mm at the neck. The patient underwent laparotomy for CBD exploration. Cholecystectomy was performed. Distal CBD was opened up, and nine leaf-like motile flatworms were found with thick bile discharge. (Fig. 1b-e) The organisms were preserved in 70% ethanol for further investigations. A single dose of oral triclabendazole (10 mg/kg) was administered. The patient was discharged in good condition 14 days after admission. On the 5th post op seven months after hospital discharge, her health condition was satisfactory.

Morphological and molecular characterization of the organisms was performed in the Department of Medical Parasitology, Kerman University of Medical Sciences. The organisms were identified as helminths belonging to the class Trematoda. The flukes were morphometrically studied, and the body length and width were measured using a digital caliper. The length and width of the flukes were 17.4–26.7 mm and 12.1–14.6 mm, respectively.

**Fig. 2:**
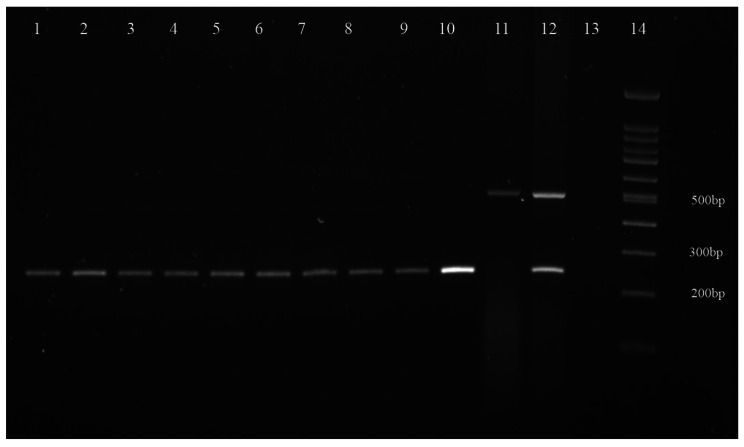
Multiplex PCR amplification of pepck gene of *Fasciola* flukes. 1–9: *Fasciola* isolates from the patient; 10–12: Control species: 10: *F. hepatica*, 11: *F. gigantica*, 12: Intermediate forms of *Fasciola*; 13: No-template control; 14: DNA size marker

For molecular identification, a small piece of the posterior parts of the flukes was cut, and DNA extraction was performed. According to previous studies, fragments of the single-copy genes phosphoenol- pyruvate carboxykinase (pepck) were amplified from nuclear DNA by multiplex PCR assay [8, 9]. The PCR products were run on a 1.5% agarose gel. (Fig. 2)

**Fig. 3:**
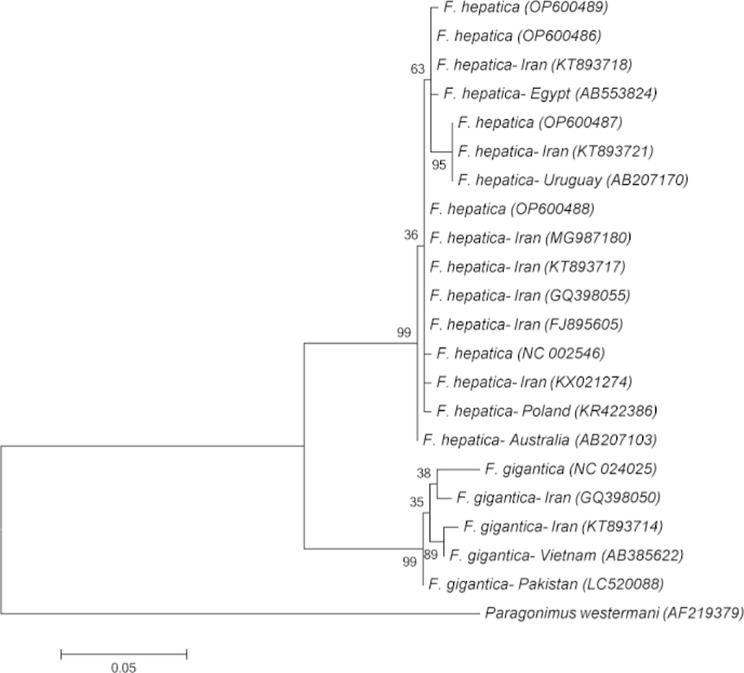
Molecular phylogenetic analysis of *Fasciola* species based on partial cox1 gene sequences from different geographical locations. Maximum Likelihood method based on Kimura 2-parameter model was used in the analysis. Four haplotypes of the *Fasciola* flukes isolated from a 42-year-old female patient were deposited in NCBI GenBank under the accession numbers, OP600486-OP600489. Evolutionary analyses were performed in MEGA-6.

For phylogenetic analysis, a 500 bp partial mitochondrial cytochrome c oxidase subunit 1 (cox1) gene fragment was amplified by Ita8: ACGTTGGATCATAAGCGTGT and Ita9: CCTCATCCAACATAACC [10]. The PCR products were sequenced by Sanger sequencing, and the sequence data were published in GenBank under the Accession Number OP600486- OP600489. Phylogenetic analysis was performed by MEGA 6.0 as described elsewhere. The phylogenetic tree was inferred by Maximum Likelihood method based on Kimura 2- parameter model with 1000 bootstrap replicates. Specific identification was also confirmed by comparison with the known sequences of the corresponding species in GenBank (Fig. 3). All the *Fasciola* isolates were identified as *F. hepatica* using both pepck multiplex PCR and cox1 sequencing. DNA sequence polymorphism analysis using DnaSP V.6.12.03 software revealed four haplotypes and five polymorphic sites in the 423 bp fragment among the nine sequenced isolates.

## Discussion

Fascioliasis is common in many parts of the world, particularly in areas where traditional livestock husbandry is a common practice. In recent years the number of human fascioliasis has increased drastically with the estimates ranging from 2.4 million to 17 million people infected [11]. Fascioliasis is endemic in many countries in Asia including Iran, Pakistan, and Afghanistan. According to 41 studies in 13 Asian countries during 2000–2015, fascioliasis among cattle has been estimated at 0.7–69.2% followed by buffaloes (2.1–68.0%), sheep (0.4–31.4%), and goats (0.0–47.0%) [12]. Sistan and Baluchestan province in southeastern Iran is neighbor to Pakistan and Afghanistan. In Pakistan, fascioliasis is mainly caused by *F. gigantica* as the predominant *Fasciola* species, and the overall prevalence in humans and livestock in the region was reported as 0.3% and 20.1%, respectively [12, 13]. Animal fascioliasis in Sistan and Baluchestsn has been investigated from 2008 to 2016. A Survey in four slaughterhouses in Sistan and Baluchestan province showed 3.1% of cattle livers were condemned due to *Fasciola* infections [14]. Lymnaeid freshwater snails, known as the intermediate hosts of *Fasciola* species have been found throughout Sistan and Baluchestan province [15], however no study has been performed to determine the extent of the snails infection with the larval stages of *Fasciola*.

Fascioliasis comprises two main stages: acute or liver phase occurs for about eight weeks when immature juvenile worm migrate from the small intestine to the liver and sometimes other organs through the abdominal cavity. At this stage, the excretory-secretory products synthesized by the fluke are responsible for abdominal pain, anemia, urticaria, diarrhea, and hyper-eosinophilia. The chronic or biliary phase begins following the acute phase during which the juveniles develop into the adult worms and release eggs. [16, 17].

In the present study, we report a human case of fascioliasis in a non-endemic region with a hot and dry climate in Southeastern Iran. This is a significant finding because the patient reports no history of traveling to endemic regions. Our knowledge of the epidemiology and transmission of fascioliasis in Iran, out of the well-known foci of the disease in the north is poor. The transmission of the disease in the north of the country follows the pattern named “Caspian pattern” with a relatively high number of human and animal cases per year with an average annual temperature and humidity of about 13.2–19.2°C and 76–88%, respectively [18]. Most human cases have been limited to the northern province of Gilan, however recently an endemic focus of fascioliasis has been reported from Kohgiluyeh and Boyer Ahmad in the southwest of Iran [19, 20]. The patient of the present report lives in a hot and dry region in southeast Iran. Another case of fascioliasis was reported from this region in 2013 in a 61-year-old man with chronic cholecystitis. During the surgery, one adult *Fasciola* worm was found in his gall bladder, however no molecular investigation was performed to identify the helminth at the species level. The patient reported consuming fresh vegetables and no history of travel to the endemic regions of fascioliasis in Iran [21]. Findings of a serological survey in Sistan and Baluchestan province indicated the presence of anti-*Fasciola* antibody in 2.4% of the subjects [22]. Our report is the second report from this region. In the north of the country, human consumption of wild vegetables such as ‘‘Chuchagh’’, ‘‘Bineh’’ and ‘‘Khalvash’’ is common, however the present patient is from an arid area with low rainfall and a dry climate. Moreover, wild vegetable consumption is not common in the region; nonetheless some of the residents consume local vegetables called Kakolak or Siahshour (*Suaeda fruticosa*), Sowzi (*Cardaria draba*), and Goshook (*Alhagi maurorum* which can be a potential source of infection. Further field studies are required to understand the epidemiological status of fascioliasis in the region.

Morphological and molecular analyses of the helminths from our patient identified the isolates as *F. hepatica*. Isolating adult worms during surgery is a rare observation in the literature [1, 23, 24]. Also, the number of parasites has varied from 1 to 12 worms in different studies [1, 24–27]. Most of the human cases of fascioliasis in Iran have been identified as *F. hepatica*. Nevertheless, the first molecular confirmation of *F. gigantica* was reported in a woman from a village near Mianeh, northwest of the country [28]. In the present study based on molecular investigation and phylogenetic analysis four haplotypes and five polymorphic sites were found among the nine sequenced isolates. The relatively high haplotype variation among the isolates from a patient can suggest that the patient has been frequently exposed to the parasite.

Right upper quadrant, fever, malaise, leukocytosis, weight loss, urticaria, anemia, epigastric pain, sweating, anorexia, jaundice, and hyper-eosinophilia are the most common presentations of human fascioliasis reported from sporadic cases from various parts of the country [18, 26]. Fascioliasis might cause acute attacks of cholangitis, cholecystitis, and biliary obstruction. The formation of stones due to fascioliasis is also possible. Acute cholecystitis due to *Fasciola* infection is sporadically reported [29]. In moderate and heavy infections mechanical obstruction of the bile ducts and gallbladder is expected due to the large body of these trematodes [26, 30–32]. The diagnosis can be supported by imaging methods. Sonography appears to be more sensitive than computed tomography in the chronic phase because some specific characters including thickening of the major bile ducts, motile or dead parasites within the ducts or gallbladder, mild dilatation, and edema of the biliary ducts are readily detected by ultrasound [33]. Conventional biliary ultrasonography in fasciolosis usually reveals dilatation and irregular thickened walls with the worms seen as vermiform structures without acoustic shadowing. Only a few reports have described the removal of living flukes (e.g., *F. hepatica*) from the biliary tract [24, 34, 35].

## Conclusion

Fascioliasis is among the etiologies of chronic cholecystitis, and physicians should consider chronic cholecystitis associated with fascioliasis in the differential diagnosis. Ultrasonography is believed to be helpful in the diagnosis of biliary fasciolosis, however it may miss distal CBD lesions. EUS is an accurate tool for exploring the lower biliary tree. As described in other studies, the role of MRCP and EUS in the biliary fasciolosis diagnosis is documented in the present report.

## Electronic supplementary material

Below is the link to the electronic supplementary material.

Supplementary Material 1

## Data Availability

Patient samples including the helminths and DNA specimens are deposited in the Department of Parasitology, Kerman University of Medical Sciences. The datasets generated and analyzed during the current study are available in the NCBI repository with the accession numbers OP600486-OP600489.

## Abbreviations

CBD: Common bile duct
PCR: Polymerase chain reaction
Pepck: phosphoenol pyruvate carboxykinase
Cox: Cytochrome c oxidase subunit 1
WHO: World Health Organization
RUQ: Right upper quadrant
WBC: White blood cells
RBC: Red blood cells
Hb: Hemoglobin
HCT: Hematocrit
MCV: Mean Corpuscular Volume
FBS: Fasting blood sugar
GGT: Gamma-glutamyl transpeptidase
AST: Aspartate aminotransferase
ALT: Alanine aminotransferase
ALP: alkaline phosphatase
MRCP: Magnetic Resonance Cholangiopancreatography
EUS: Subsequently Endoscopic Ultrasound
DNA: Deoxyribonucleic acid
ELISA: Enzyme-linked immunosorbent assay
