# Supplementary figures and images for: Fascioliasis associated with chronic cholecystitis in a woman from Sistan and Baluchestan province, a non-endemic region in Southeastern Iran
Source: BMC Infect Dis. 2023 May 19;23:336. doi: 10.1186/s12879-023-08310-z (PMC10199601; doi:10.1186/s12879-023-08310-z)

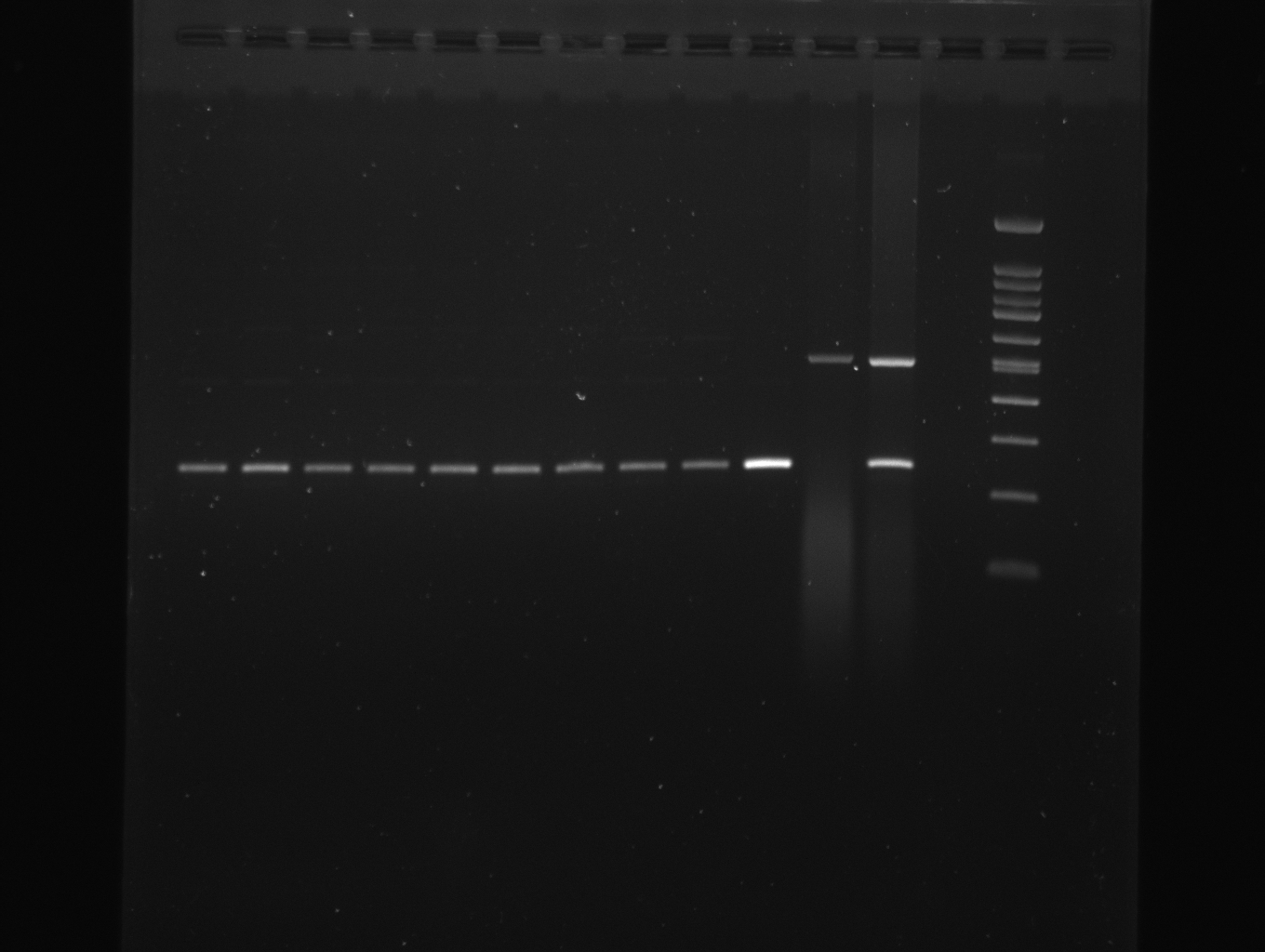

Supplement: Supplementary file 1 — Supplementary Material 1 [file 12879_2023_8310_MOESM1_ESM.tif]
